# Out-of-Pocket Expenditure on Medical Services Among Older Adults: A Longitudinal Analysis

**DOI:** 10.3389/fpubh.2022.836675

**Published:** 2022-04-07

**Authors:** Aviad Tur-Sinai

**Affiliations:** ^1^Department of Health Systems Management, The Max Stern Yezreel Valley College, Yezreel Valley, Israel; ^2^The Minerva Center on Intersectionality in Aging, University of Haifa, Haifa, Israel

**Keywords:** health insurance, out-of-pocket, forgone medical care, old age, private funding, SHARE

## Abstract

The upturn in life expectancy and its consequence, population aging, are challenging labor, pension, and social-security systems throughout the developed world. The focal aim of this study is to measure the extent of out-of-pocket funding of healthcare services by the older adult population. The study has three objectives: to profile the healthcare services for which older adults pay out of pocket, profile the older adults who pay out of pocket for medical services and detect changes over the years, and identify predictors of out-of-pocket healthcare services funding by older adults. The study is predicated on the SHARE-Israel database (SHARE—Survey of Health, Ageing and Retirement in Europe). Relating to information yielded by the last two waves of SHARE-Israel (Wave 7 and Wave 8), it sheds light on the characteristics of those who reported having paid out-of-pocket for medical services. A large majority of the older-adult population in Israel that consumes healthcare services is asked to pay for services out of pocket. Having supplemental health insurance, personal state of health and changes in it, and economic resources are found to have the strongest effect on the probability of out-of-pocket funding. The motive of financial and/or social support that older adults receive from and/or give to their immediate surroundings makes it more likely that they will pay out of pocket for healthcare services. The probability of such funding varies between nationalities and immigration statuses. It is found with emphasis that the share of out-of-pocket funding of healthcare in older adult households' total annual income is trending upward. Furthermore, economic motives are central in determining whether such expenditure will stabilize over time. The findings stress the need to enhance the healthcare system's awareness of the profile of older adults who find it necessary to pay out of pocket for healthcare services.

## Introduction

Rising life expectancy and population aging are global phenomena (1) that are challenging societal arrangements throughout the developed world. Almost all societies are affected today, and will be affected in the future, by changes in their populations' age distribution as the share of the young falls and that of the old rises (2). Although Israel is one of the youngest countries in the OECD (founded in 1948), its older-adult population is expected almost to double between 2010 and 2035 (3). According to its demographic forecasts, based on population estimates at the end of 2010 and foreseen developments out to 2035, the share of those aged 65+ in the population will climb to 12.3% in 2010, 13.9% in 2030, and 14.6% in 2035 (the middle alternative in the forecast) (4). The dependency ratio of the elderly (the ratio of those aged 65+ to those aged 15–64) is projected to continue climbing steadily in coming decades. This trend will oblige the state to contend with its economic implications: a foreseen upturn in expenditure on funding of the social safety net and healthcare and nursing services for the elderly, even as proportionately fewer and fewer people will be there to bear the funding burden.

Healthcare is a basic right. International law entitles every individual and household to a standard of living high enough to assure health and welfare, including requisite medical care and security in the event of illness (5). Every healthcare system aspires to improve the population's health while maintaining equality, access, and satisfaction among consumers and striving to enhance effectiveness (6). Two goals of Israel's State Health Insurance Law (1994) are to improve equality in healthcare among population groups and deliver better service to disadvantaged groups such as minorities, residents of peripheral areas, and the elderly (7). The statute established the universal legal entitlement of all residents to healthcare services (8). It also reduced health-insurance rates for persons aged 65+ relative to those paid by the elderly before the law went into effect (9).

Israel's forecast for healthcare expenditure on the 65+ group, presented in discussions at the 12th Dead Sea Conference on Health Policy, shows that while public healthcare expenditure is expected to increase by 149% between 2010 and 2030 (7.1% on average per year), private healthcare spending is poised to grow by 168% by then (8% p.a.) (10). This means an increase in the share of private funding in disposable income, assuming that disposable income continues to grow as quickly as it has in previous years. Observing these trends, the conference participants found the current mix of private funding inconsistent with the principles of the State Health Insurance Law and advised that the upward trend in private funding of healthcare and nursing expenditure by the elderly, and of private funding all told, should be cut back.

To implement this recommendation, decision-makers need to get a full picture of the relevant information on the topic, one that will complement what is already known today. Therefore, this study centers on the problem of private funding of healthcare services among the elderly.

Israel's State Health Insurance Law, effective January 1, 1994, predicates national health insurance on “principles of justice, equality, and mutual aid” (State Health Insurance Law, 1994). To bring this about, a basket of healthcare services, to which all residents would be entitled efficiently and equally, was set forth. Freedom of choice and the right to switch among health funds (public healthcare-service providers, akin to HMOs) was also determined, as was the charging of a health-insurance fee that would be collected by the National Insurance Institute. HMOs were also allowed to offer their members a menu of supplemental insurance policies that would cover healthcare services outside the basket.

After the State Health Insurance Law was enacted, one might have expected health inequalities among population groups in Israel to narrow. The findings show that this did not happen: the health gaps have not been shrinking (11). In Israel, one finds disparities in life expectancy and physical and mental morbidity among population groups differentiated by socioeconomic status, sectorial affiliation, and other parameters. Each decline in socioeconomic status is associated with an increase in the risk of defective health (12).

## The Structure of Healthcare System Funding

In a large majority of OECD countries, national healthcare expenditure as a share of Gross Domestic Product (GDP) has been trending upward due to population aging, technological improvements that are making medical procedures increasingly expensive, and rising standards of living, along with the spread of consumer behavior patterns vis-à-vis the healthcare industry (13). In Israel, the picture is different. Since the State Health Insurance Law was passed in 1995, the fraction of expenditure on medical services in GDP has been stable and the disparity in healthcare expenditure between Israel and the rest of the OECD constituency has been widening. Thus, while average national healthcare expenditure in OECD countries is 8.9% of GDP, that in Israel was 7.5% in 2013 (14).

Healthcare systems get their funding from two sources: public and private. Public funding includes all sources that originate in the state budget and earmarked taxes, including health-insurance contributions. Private funding comprises household outlays for healthcare services, whether the services are included in national health insurance or not (15). Healthcare systems that obtain most of their funding from public sources have become mainstays in all welfare states, expenditure on them rising with each passing year (16). A healthcare system that is funded solely from public sources (i.e., deriving all of its revenues from taxes) may impose a heavy burden on taxpayers and create a disincentive for family support of elderly members who need nursing care (17). A system that relies exclusively on private funding, in contrast, is unfair to the socioeconomically disadvantaged: it widens health inequality, makes healthcare services less accessible, and erodes human capital, an important factor in economic growth (18). Therefore, an optimum mix of these two funding methods needs to be found.

The scientific literature provides four main models for structuring the public vs. private funding of healthcare expenditure. In the first model, parallel funding, some healthcare services have a private funding system in place that is a (full) alternative to public funding (19). In the second, the copayment model, healthcare services are partly funded by users or private health insurance (20). Wherever this model is invoked, socioeconomically disadvantaged populations may forgo vital medical treatments. The third model, group-based funding, entitles specific groups to public funding and allows others to access the system via private insurance (21). This model is typical of the Netherlands, where the level of public funding exceeds the OECD norm by far. Where such a model is used, regulation is needed to maintain fixed standards of medical service among groups (22). The fourth model, funding per type of service, gives some sectors of the healthcare system full private funding and leaves others to pay their own way (23). This model is used, for example, in Canada, where most inpatient services receive full public funding but most ambulatory services rely on private funding.

The structure of funding in Israel's healthcare system is not unequivocally consistent with any of these four models. It assures a comprehensive basket of healthcare services that are funded largely from public sources (but include copayments), along with other services funded by households either directly or via private health insurance. This structure enhances access to healthcare services, foremost for socioeconomically disadvantaged populations, and mitigates the risk to households of future expenses that will devastate their standard of living (18, 24).

Since Israel's State Health Insurance Law went into effect, public funding of healthcare has been eroding and the share of private funding in national healthcare expenditure has been rising steadily. This is consequent to two interrelated developments: erosion of public funding sources and acceleration of the upward movement of private expenditure (Dead Sea Conference, 2011). In 2013, public funding accounted for 61.0% of total expenditure, as against 67.4% on the eve of the healthcare-system reform in 1995. On average among the OECD countries, in contrast, the share of public funding in total healthcare expenditure is 73% (25).

In Israel, total privately funded healthcare expenditure comes to 3% of GDP. Expenditure on private insurance is 0.82% of GDP, as against only 0.45% on OECD average and 5.24% in the United States. The rate of out-of-pocket expenditure in Israel is much higher, at 2.05% of GDP as against 1.47% on OECD average and 1.80% in the U.S. Consequently, equality in Israel is suboptimal, its share of out-of-pocket spending exceeding the OECD average and even the U.S. level (26). In this context, it is noteworthy that 82.9% of Israel residents have private insurance (either commercial or through HMOs) as against 36% on OECD average, making Israel's rate the third-highest among members of the Organization (14).

The regressivity of Israel's funding of medical services finds expression in household budgets. The increase in private funding for the healthcare system is mitigating access to medical services among weak population groups by lessening their ability to afford the rising prices and reducing the availability of the services themselves (27). It is also exacerbating disparities in disposable-income distribution and contributing to an upturn in poverty among groups that are disadvantaged to begin with (28). The extent and depth of poverty differ from one society and group to another, but all have several commonalities—mainly a shortage of economic resources that results in inability to consume basic goods and service, usually accompanied by manifestations of misery, humiliation, harsh living conditions, poor health, and sometimes even social exclusion (29).

## Healthcare Costs for Adults

Even though many countries have taken steps in recent decades to improve the economic welfare of their adult populations, this population still has substantial pockets of poverty, foremost among elderly women (30–32). A study on long-term poverty found this phenomenon to be more salient in the 50–64 age group than among those aged 65+ and more evident among those who reported a downturn in their state of health than among others (33).

Morbidity rates rise with age, many older adults reporting having been diagnosed with illness (34). Older adults' patterns of healthcare service consumption are singular mainly due to the types of illnesses that they incur and their levels of expenditure on frequent needed medical care and recourse to protracted inpatient services. The frequency of chronic illnesses rises with age and such illnesses, in greater part, are not followed by convalescence and are often accompanied by functional limitations (35). In a challenge to the conventional wisdom, Hooyman and Kiyak (36) find that older adults come down with seasonal illnesses less frequently than young people do. When an older adult has a seasonal illness, however, he or she takes longer to recover and is more at risk of complications if not death. The cost of lengthy treatment is expected to increase in coming years due to population aging. This cost includes institutional inpatient care, home care, and loss of work days due to care for an older adult family member by the older adult's main source of (informal) support (37).^1^

According to De Nardi et al. (39), out-of-pocket medical expenses rise with age. Shmueli (40) adds that out-of-pocket spending on medicines does the same. In an analysis based on the American Health and Retirement Study (HRS), McGarry and Schoeni (41) find that out-of-pocket expenditure is especially onerous at end of life and is largest relative to income among the low-income elderly. Goda et al. (42) show that out-of-pocket expenditure increases by 29% on average after one is widowed and that much of this expenditure goes for at-home nursing care. The economic burden that falls on the household may prove, after the fact, definitive in deciding whether to continue making this expenditure or forgoing service (43, 44). A relation may also exist between the characteristics of an elderly individual's social structure and relationship and his or her immediate surroundings and personal wellbeing and health (45, 46).

## The Research Problem and Main Goals

The forecast of healthcare expenditure in Israel among the 65+ population shows that while public expenditure is poised to rise by 64% between 2022 and 2030 (7.1% on annual average), private spending on healthcare will grow by 72% during that time (8% on annual average). This portends an increase in the burden of private funding as a share of disposable income, assuming that disposable income will continue to increase at the rate typical of previous years. In view of these trends, it has been stated that *the current mix of private funding does not square with the principles of Israel's State Health Insurance Law, that the upward trend in private funding of healthcare and nursing services for the elderly needs to be slowed, and that private funding overall should be cut back* (10).

Surprisingly, the road to implementing these recommendations evidently remains long. To correct this, healthcare-system policymakers need to be fully equipped with relevant information on the topic, complementing what is known thus far. Three main fields of knowledge are deficient: the medical treatments that older adults fund out of pocket, the identity of the older adults who fund their healthcare in this manner, and the predictors of out-of-pocket funding of healthcare services by people in the second half of their lives. Contending with these three areas of inadequate knowledge is the underlying aim of this study.

The study centers on the question of the extent of out-of-pocket expenditure on healthcare services among the elderly. The increase in private funding of the healthcare system is aggravating poverty among weak population groups and impairing these groups' access to vital services. The prolongation of life expectancy, the increase in older adults' dependency ratio, and the forecast of continued upturns in the burden of private funding as a percent of older adults' disposable income raise concerns about far-reaching implications for the welfare and healthcare system that serves the elderly population.

Three research questions, each independent of the others, underlie this study. The mosaic of information obtained by answering them will fill a major lacuna in understanding the matter of out-of-pocket funding of healthcare services for the elderly in Israel. The three questions are:

1. What are the characteristics of the healthcare services for which the elderly pay out-of-pocket?
2. Who are the elderly who pay for healthcare services out-of-pocket?
3. What factors predict out-of-pocket funding of healthcare services among people in the second half of their lives?

## Methods

### Research Population and Databases Used

The study is based on the SHARE-Israel database (SHARE— Survey of Health, Ageing and Retirement in Europe). SHARE is a multinational (European) panel study comprised of representative samples of the population aged 50+ in multiple countries.

SHARE aspires to create a better understanding of the situation of the growing and substantial population of persons aged 50+ and construct a framework for the development of research infrastructures for public policy-making vis-à-vis this population (47). The information gathered in SHARE constitutes a unique opportunity to compare the health, economic situation, and welfare of older adults in various European countries from an intertemporal and multidisciplinary perspective that facilitates cross-country analyses in a broad range of matters at different periods and lengths of time (48). The survey provides extensive information about individuals' health and functioning, household structure, employment, financial transfers, household income, economic expectations, and quality of life (49). It illuminates the characteristics of those who reported having had to pay out-of-pocket for medical treatments. SHARE is congruent with international surveys such as the American HRS and the English Longitudinal Study of Ageing (ELSA). Similarly structured surveys are under way in Japan, China, India, and Brazil (50).

We use data from the last two waves of SHARE—Wave VII (2017) (hereinafter referred to as Time 1 or the first investigation period) and Wave VIII (2019/2020) (hereinafter Time 2 or the second investigation period). The study relates to data harvested from these two waves in their reference to Israel only.

### Research Variables

#### Explained Variables

To investigate out-of-pocket spending on medical treatments/medicines in Israel, five research variables were defined. The first relates to those who received a treatment in an inpatient setting in the 12 months preceding the day of their interview—**out-of-pocket expenditure on inpatient care**. The variable is based on the question, “Not counting your health-insurance premiums and employer's reimbursement of expenses, approximately how much did you pay out of pocket for all treatments that you received in inpatient settings in the past twelve months?” The second variable pertains to those who received care in outpatient clinics in the 12 months preceding the date of the interview—**out-of-pocket expenditure on outpatient clinics**. It is based on the question, “Not counting your health-insurance premiums and employer's reimbursement of expenses, approximately how much did you pay out of pocket for all treatments that you received in outpatient settings in the past twelve months?” The third variable relates to those who received prescription medicines in the 12 months preceding the date of the interview—**out-of-pocket expenditure on prescription medicines**. It is based on the question, “Not counting your health-insurance premiums and employer's reimbursement of expenses, approximately how much did you pay out of pocket for all prescription medicines that you received in the past twelve months?” The fourth variable pertains to those who received caregiving services in nursing institutions, day centers, and/or at home in the 12 months preceding the interview date—**out-of-pocket expenditure on treatments at nursing institutions/at-home caregiving services**. This variable is based on the question, “Not counting your health-insurance premiums and employer's reimbursement of expenses, approximately how much did you pay out of pocket for all treatments that you received in nursing institutions and day centers and for all at-home caregiving services in the past twelve months?” All four variables described above are dichotomous, based on two values, i.e., an out-of-pocket expenditure on the care at issue was made or was not made. The fifth and final variable addresses those who received one or more of the medical treatments itemized above in the 12 months preceding the interview date; it asks whether there was an **out-of-pocket expenditure on any healthcare service** during that time. This variable is also dichotomous: either there was an out-of-pocket expenditure on healthcare services or there was not.^2^ The data of the explained variables were collected at Time 2.

#### Explanatory Variables

The group of explanatory variables that yield a profile of, and predict, out-of-pocket funding for healthcare services among people in the second half of their lives should is mapped into four sub-groups. The data of all explanatory variables were collected at Time 1, except of two health change variables which were constructed by subtracting the values in Time 2 from those of Time 1.

The first sub-group is defined by *sociodemographic characteristics* and amalgamates individuals' age, gender, education, nationality, and immigration, as well as religiosity (measured on the basis of frequency of attending worship services).

Numbered among the second sub-group of explanatory variables are those relating to the respondents' *social network* (interpersonal environment). A response about the connection between the characteristics of an older adult's social network and his or her out-of-pocket expenditure on healthcare services is elicited by using several variables that are familiar from the literature on the topic (52): living with others (a dichotomous variable that has two outcomes: 1 if the respondent reports living with others, 0 otherwise), household size, social support, and financial support. The social-support component is based on two paths. The first is giving social support (a dichotomous variable that has two outcomes: 1 if the person reports having received help of some kind from a family member/friend/neighbor in the 12 months preceding the interview date, 0 otherwise. The financial-support component is based on two paths. The first is giving financial support (a dichotomous variable that has two outcomes: 1 if the person reported having bestowed a financial or material gift, or support of some kind, on a member of his or her household or on someone else in the 12 months preceding the interview date; 0 otherwise); the second is receiving financial support (a dichotomous variable that has two outcomes: 1 if the person reported having received a financial or material gift, or support of some kind, from a member of his or her household or from someone else in the 12 months preceding the interview date; 0 otherwise).

The third sub-group of explanatory variables relates to *health*. Participants' self-rated health includes a wide variety of variables such as number of chronic illnesses, increase in the number of chronic illnesses, self-rated health (1 = excellent, 5 = poor), worsening of self-rated health, and the EURO-D depression symptoms index.

The fourth and final sub-group of variables amalgamates information about *economic resources and quality of life*. Included among these variables are employment (a dichotomous variable that has two outcomes: 1 if a person reports being employed; 0 otherwise) and household income (from labor and other sources). To investigate the presence or absence of a connection between having supplemental health insurance and spending out of pocket on healthcare services, a separate research variable—supplemental health insurance—was defined. It is a dichotomous variable that has two outcomes: a respondent either purchased supplemental health insurance from his or her HMO or did not. The quality-of-life indicators are satisfaction with life (0 signifying “totally dissatisfied” and 10 denoting “totally satisfied”) and living in a peripheral area (a dichotomous variable that has two outcomes: 1 if the person reports living in a location on the periphery, 0 otherwise. This definition is based on the peripherality index of the Israel Central Bureau of Statistics in accordance with the individual's locality of residence.

#### Data Analysis Methods

The data were analyzed using STATA Version 15.1 (53). First, a statistical profile of the frequency of out-of-pocket expenditure on healthcare services and the level of expenditure was produced, as was a profile of the socio-demographic background characteristics, health characteristics, and sources of financial wherewithal of those who spent out of pocket on healthcare services. This was followed by an econometric estimation. The first econometric model focused on determining predictors of out-of-pocket healthcare expenditure among older adults. It was performed by estimating a Logit model, in which the marginal effect of each estimated variable was presented. To complete the picture, an additional econometric model was estimated using a multinomial technique. This model tested for the probability of transitions among situations of out-of-pocket expenditure on healthcare services during the two investigation periods.

## Results

The large majority of respondents—persons aged 50+ who received healthcare services—paid some amount out of pocket for such services, in addition to their fixed compulsory health-insurance contribution and reimbursement of expenses by employers (85% at Time 1, 89% at Time 2) (Table 1). Some 24% of respondents who received inpatient care at Time 1 paid out of pocket for such care; a similar proportion did so at Time 2. Some 42% of those who received care in outpatient clinics at Time 1 paid something for it; a similar proportion (46%) did so at Time 2. More than 80% of older adults who received prescription medicines paid something out of pocket for them, beyond their compulsory health-insurance contributions or reimbursement of expenses by employers. Furthermore, 20% of respondents who received care in nursing institutions, day centers, or at home in the year proceeding Time 1 paid something out of pocket for it. Two years later, the share of out-of-pocket spending for care in nursing institutions, day centers, or at home among the elder population was 33%.

**Table 1:** Out-of-pocket expenditure on healthcare services among recipients of healthcare services aged 50+ in investigation time 1 and 2, panel (Pct.).

	**Out-of-pocket spending for**				
	**Inpatient care**	**Outpatient clinic care**	**Prescription medicines**	**Nursing-institution/at-home care**	**Healthcare services**
Time 1	24.39	42.04	81.33	21.85	85.19
Time 2	22.19	46.37	85.8	32.55	89.87
Significance	0.19	0.01	0	0	0

The average annual out-of-pocket expenditure on healthcare services among elders who received such services in Israel was € 809 at Time 1 and € 832 at Time 2−4.5% of total annual household income at Time 1 and 5.2% 2 years later (Table 2). The average annual out-of-pocket expenditure on inpatient care for older adults was € 562 in Time 1 and about half as much 2 years later−4.2% of total household annual income at Time 1 and roughly half of this fraction 2 years later. The average annual out-of-pocket expenditure on outpatient-clinic services was € 326 in Time 1; that on prescription medicines was the same—~2.8% of total annual household income at Time 1 in both cases. Two years later, the average annual out-of-pocket expenditure on outpatient care was € 373, 3.3% of total annual household income, and the average annual out-of-pocket expenditure on prescription medicines was roughly € 411, 3% of total household annual income. The average annual out-of-pocket expenditure on care in nursing institutions or at home was € 763 at Time 1, 2.7% of total annual household income, and € 996 and 4%, respectively, 2 years later.

**Table 2:** Characteristics of out-of-pocket expenditure on healthcare services among recipients of healthcare services aged 50+ at investigation time 1, panel^a^ (€, 2020 prices).

	**Out-of-pocket spending for**				
	**Inpatient care**	**Outpatient clinic care**	**Prescription medicines**	**Nursing-institution / at-home care**	**Healthcare services**
**Time 1**					
Mean	561.95	325.73	312.47	763.31	809.04
S.D.	2,644.43	1,277.02	452.43	2,362.95	1,997.33
Minimum	0	0	0	0	0
Maximum	33,783.78	20,270.27	5,405.41	21,081.08	34,864.86
Pct. of total income	4.19	2.79	2.84	2.72	4.54
**Time 2**					
Mean	315.15	372.81	410.93	996.29	832.05
S.D.	306.19	1,390.20	543.65	4,022.05	2,063.95
Minimum	0	0	0	0	0
Maximum	2,702.70	35,135.14	6,756.76	38,919.92	38,919.92
Pct. of total income	1.96	3.28	2.98	4.01	5.17

a *Proportion of total income pledged to out-of-pocket expenditure on healthcare services not covered by insurance policies and plans*.

The average age of elders who incurred out-of-pocket expenses for healthcare services was sixty-seven. Those who spent out of pocket for inpatient care were ~72 years of age and those who incurred out-of-pocket expenses for nursing-institution or at-home caregiving services were 78.5 on average (Table 3). More than two-thirds of those who spent out of pocket for healthcare services reported that they were living with others, except for those who spent personally on nursing-institution or at-home care—among whom only 27% lived at home. The average proportion of men among elders who spent out of pocket for care was 42%, and a similar percentage was found in regard to each of the types of out-of-pocket expenditure except nursing-institution or at-home care services, in which men were on average only 14% of those who spent out of pocket. Some 8% of older adults who spent out of pocket for healthcare services lived in peripheral areas.

**Table 3:** Background, health, and economic-resource variables among persons aged 50+ who paid out of pocket for healthcare services at investigation time 1^a^^b^^c^.

		**Out-of-pocket spending for**					
		**Inpatient care**	**Outpatient clinic care**	**Prescription medicines**	**Nursing-institution/at-home care**	**χ2/*t*** **(*P*. Value)**	**Healthcare services**
Age (years)		71.89 (9.72)	66.67 (9.65)	67.24 (9.97)	78.56 (9.76)	<0.01	67.10 (9.94)
Male (%)		41.43	43.62	42.17	13.85	<0.01	42.71
Education (years)		10.10 (5.73)	11.85 (4.71)	11.44 (4.86)	8.94 (4.75)	0.019	11.53 (4.89)
Jewish (%)		95.53	92.08	88.97	94.73	0.046	89.35
Born in investigation country (%)		92.83	92.68	90.86	90.84	0.088	91
Frequency of worship (%)	>1 per day	20.16	13.94	17.06	20.41	<0.01	16.63
	Once per day	12.45	7.9	9.44	2.77		9.21
	Several times per week	4.94	2.54	2.65	0.58		2.65
	Once per week	9.81	11.93	10.37	14.88		10.37
	<1 per week	4.26	15.46	13.87	17.21		14.03
	Never	48.38	48.23	46.61	44.14		47.1
Lives with others (%)		64.61	71.47	71.06	26.57	0.018	71.4
Size of household		2.42 (1.48)	2.09 (0.96)	2.14 (1.05)	2.15 (1.37)	0.023	2.13 (1.03)
Receives social support (%)		66.35	32.42	30.84	73.2	<0.01	30.27
Gives social support (%)		17.52	25.81	25.25	8.44	<0.01	25.63
Receives financial support (%)		5.01	7.57	8.49	13.37	0.008	8.69
Gives financial support (%)		38.43	48.95	45.54	28.49	0.012	45.95
Chronic illnesses (*N*)		3.08 (1.92)	1.98 (1.76)	2.03 (1.71)	3.51 (1.84)	<0.01	2.94 (1.71)
Change in chronic illnesses (1)		0.78 (1.99)	0.49 (1.73)	0.53 (1.76)	1.23 (2.53)	<0.01	0.51 (1.75)
Self-rated health		4.34 (0.89)	3.42 (1.10)	3.47 (1.09)	4.46 (0.73)	0.014	3.73 (1.11)
Worsening in self-rated health		35.96	41.48	39.31	36.78	0.026	39.4
EURO-D		3.94 (2.86)	2.73 (2.54)	2.86 (2.58)	4.22 (2.76)	0.017	2.83 (2.56)
Employment (%)		11.84	35.23	31.91	1.22	<0.01	33.13
Net income (€, 2020 prices)		2,678.18 (2,637.52)	3,342.14 (3,106.64)	3,126.01 (3,009.03)	1,870.53 (2,064.92)	<0.01	3,190.50 (3,054.52)
Has supplemental health insurance (%)		72.44	86.17	84.05	88.25	0.026	84.44
Satisfied with life		4.57 (3.95)	6.82 (3.15)	6.69 (3.18)	2.77 (4.06)	<0.01	6.72 (3.18)
Lives in peripheral area		10.2	9.9	7.76	6.67	0.013	7.86

a *Weighted on the basis of weights at Time 2*.

b *Values in parentheses are Standard Deviations*.

c *The data relate to Time 2 among members of the panel*.

Those who spent out of pocket for outpatient care and prescription medicines had the fewest chronic illnesses, two on average. Those who spent in this manner for inpatient nursing care or other caregiving services had the largest number of chronic illnesses during the investigation period, 3.5, and the largest average increase, 1.2.

The average self-rated health was highest among those who spent out of pocket for inpatient services, care in nursing institutions, or other caregiving services (4.5 out of 5). Furthermore, nearly 40% of those who spent out of pocket for healthcare services reported a decline in their state of health during the investigation period.

Satisfaction with life was uneven among those who spent out of pocket for healthcare services. It was highest among those who spent on outpatient-clinic services and prescription medicines. Some 84% of respondents who incurred out-of-pocket expenses for healthcare services had supplemental health insurance. A similar share was found for each group of out-of-pocket expenditure except those who spent for inpatient care, of whom only 72% had supplemental health insurance.

As for the social-network indicator among the four types of out-of-pocket expenditure on healthcare services, variance was found in the characteristics of social support and financial support, particularly when out-of-pocket expenditure on nursing-institution/home-care services was compared with the other types of out-of-pocket spending on healthcare services. This divergence recurred in regard to both economic indicators, income and employment. For them, it was found that the net income of a household that spends out of pocket for inpatient/outpatient care or for prescription medicines is almost twice that of households that spent out of pocket for nursing-institution/at-home care. Furthermore, the rate of employment was only 1% among those who incurred this kind of expenditure as against 12% among those who spent out of pocket for inpatient care and a steep 30% among those who spent out of pocket for outpatient-clinic care or prescription medicines.

Thus far, the study has contended with the first two research questions: What are the characteristics of the healthcare services for which the elderly pay out-of-pocket, and who are the elderly who pay for healthcare services out-of-pocket? Now the third question is discussed—what factors predict out-of-pocket funding of healthcare services among people in the second half of their lives? The answers are sought via a range of two-choice and multiple-choice models that take the structure of the panel and changes over time into account.

The probability of out-of-pocket funding of healthcare services among people in the second half of their lives is investigated in Table 4. Model 1, focusing on analysis of the sociodemographic characteristics, finds that the probability of out-of-pocket funding rises with the individual's age. Thus, the likelihood of out-of-pocket funding is 5.4% points higher among those aged 65–79 than among those aged 50–64 and 6.5% points higher among those aged 80+. The probability correlates negatively with education (each year of schooling lowers it by 3.35 points). The probability of out-of-pocket funding is 1.8% points lower among Arabs, and 1.5% lower among former Soviet immigrants, than among non-immigrant Jews. Furthermore, the probability of out-of-pocket funding of healthcare services is negatively dependent on the frequency of attending worship services.

**Table 4:** Probability models for out-of-pocket expenditure on healthcare services among persons aged 50+ at investigation time 1, panel (marginal effect).

		**Model 1**	**Model 2**	**Model 3**	**Model 4**	**Model 5**	**Model 6**
Sociodemographic indicators	**Age (baseline: 50–64)**						
	65–79	0.054^***^	0.058^***^	0.052^***^	0.026^**^	0.022^**^	0.019^**^
	80+	0.065^***^	0.064^***^	0.058^***^	0.036^**^	0.031^**^	0.027^**^
	Gender (baseline: female)	−0.016	−0.016	−0.009	−0.009	−0.01	−0.006
	Education (years)	−0.033^***^	−0.029^***^	−0.029^***^	−0.028^**^	−0.028^**^	−0.019^**^
	**Nonimmigrant Jews**						
	Arabs	−0.018^**^	−0.014^**^	−0.011^**^	−0.010^**^	−0.007^*^	−0.007^*^
	FSU immigrants	−0.015^***^	−0.010^**^	−0.009^**^	−0.009^**^	−0.009^**^	−0.011^**^
	**Frequency of worship (baseline: more than once per day)**						
	Once per day	0.036^**^	0.036^**^	0.035^*^	0.027^**^	0.017^**^	0.013^*^
	Several times per week	0.006	−0.003	0.003	−0.001	0.007	0.015
	Once per week	0.049^***^	0.049^***^	0.043^***^	0.035^***^	0.020^**^	0.013^*^
	Less than once per week	0.065^***^	0.067^***^	0.058^***^	0.042^***^	0.026^***^	0.019^***^
	Never	0.056^***^	0.059^***^	0.058^***^	0.046^***^	0.032^***^	0.021^**^
Social network	Lives with others (baseline: lives alone)		0.026^**^	0.031^***^	0.029^**^	0.024^**^	0.018^**^
	Size of household		−0.019^**^	−0.013^**^	−0.015^**^	−0.017^**^	−0.014^**^
	Gives social support			0.018^**^	0.013^**^	0.008^*^	0.006^*^
	Receives social support			0.035^***^	0.022^**^	0.019^**^	0.021^**^
	Gives financial support			0.024^***^	0.021^***^	0.023^***^	0.028^***^
	Receives financial support			0.019^**^	0.015^**^	0.017^**^	0.014^**^
Health	No. of chronic illnesses				0.035^***^	0.040^***^	0.036^***^
	Increase in no. of chronic illnesses					0.029^***^	0.025^***^
	Self-rated health				0.019^***^	0.018^***^	0.014^**^
	Worsening in self-rated health					0.009^**^	0.012^**^
	EURO-D				−0.004	−0.004^*^	−0.002^*^
Economic resources & quality of life	Employment						0.023^**^
	Household income (In)						0.008^**^
	Supplemental health insurance						−0.042^***^
	Satisfaction with life						0.001
	Lives in periphery						0.022^***^
Pseudo *R*^2^		0.0398	0.0452	0.0526	0.1487	0.2384	0.2678

*Source: SHARE-Israel*.

* *P < 0.1;*

** *P < 0.05;*

*** *P < 0.01*.

After the sociodemographic indicators were mapped and were found to play a role in the probability of out-of-pocket funding of healthcare services among the elderly, the model examined the effects of the remaining elements on this decision. First investigated was the connection attributed to the social network in this decision, presented in Model 2. The results of the model show that the probability of out-of-pocket funding of healthcare services is 2.6% points higher among respondents who live with others, each additional individual in the household lowering the probability by 1.9% points.

Model 3 adds social-network indicators that relate to social and financial support. The analysis shows that giving social support raises the probability of out-of-pocket funding of healthcare services by 1.8% points, receiving social support increases the probability by 3.5% points, giving financial support elevates it by 2.4% points, and receiving financial support increases the likelihood of out-of-pocket funding of healthcare services among the elder population by 1.9% points.

Model 4 contributes to and examines the question of whether an older adult's state of health has something to do with his or her decision to pay out of pocket for healthcare services, and it maps the relative effect of the various factors in this context. The results of the model show that the probability of out-of-pocket funding rises with the number of chronic illnesses that a person has (a marginal effect of 3.5% points) and with an increase in the individual's self-rated health (a marginal effect of 1.9% points).

Model 5 adds an aspect on the health side, asking whether long-term changes in health affect the probability of spending out of pocket for healthcare services. The results of the model show that an increase in the number of chronic illnesses raises the probability of out-of-pocket funding of healthcare services by 2.9% points, whereas a worsening in self-rated health acts in the same direction by 0.9% point.

Model 6 augments everything done thus far by examining the effects of economic resources and quality of life. The analysis shows that the likelihood of spending out of pocket for healthcare services is more than 2.3% points greater among employed older adults than among unemployed ones and that household income is a significant consideration in the decision on whether to spend out of pocket for healthcare services. Having supplemental health insurance lowers the probability of spending out of pocket for healthcare services by 4.2% points, and the probability of out-of-pocket expenditure on healthcare services is 2.2% points greater among those who live in peripheral areas than among those who do not.

Notably, the use of an OLS model to estimate the average annual out-of-pocket expenditure for healthcare services among older adults who receive healthcare services yielded results consistent with those presented above. In other words, the factors that explain the probability of spending out of pocket for healthcare services were also found in the present context and the direction of the effect of the estimated variable remained unchanged.

One of the most interesting issues in regard to spending out of pocket for healthcare services is the role of supplemental insurance. Thus far, as stated, it has been found that having such insurance lowers the probability of spending out of pocket for healthcare services. Table 5 presents the findings of a model of the probability of an elder's spending out of pocket for healthcare services as a function of having supplemental insurance.

**Table 5:** Probability models for out-of-pocket expenditure on healthcare services among holders of supplemental insurance aged 50+ in investigation time 1, panel (marginal effect).

		**Has supplemental health insurance**	
		**Yes**	**No**
Sociodemographic characteristics	**Age (baseline: 50–64)**		
	65–79	0.018^**^	0.016^**^
	80+	0.025^**^	0.029^***^
	Gender (baseline: female)	−0.004	−0.010
	Education (years)	−0.017^**^	−0.014^**^
	**Non-immigrant Jews**		
	Arabs	−0.004^*^	−0.007^***^
	FSU immigrants	−0.008^**^	−0.013^***^
	**Frequency of worship (baseline: more than once per day)**		
	Once per day	0.013^**^	0.015^**^
	Several times per week	0.015^*^	0.010
	Once per week	0.013^**^	0.015^**^
	Less than once per week	0.014^**^	0.024^**^
	Never	0.016^**^	0.028^***^
Social network	Lives with others (baseline: lives alone)	0.022^**^	0.024^**^
	Size of household	−0.021^**^	−0.018^**^
	Gives social support	0.004^*^	0.006^*^
	Receives social support	0.019^**^	0.017^**^
	Gives financial support	0.033^***^	0.029^***^
	Receives financial support	0.010^*^	0.024^***^
Health	No. of chronic illnesses	0.038^***^	0.044^***^
	Increase in no. of chronic illnesses	0.020^***^	0.027^**^
	Self-rated health	0.013^**^	0.015^**^
	Worsening in self-rated health	0.008^**^	0.012^***^
	EURO-D	−0.002	−0.008^*^
Economic resources & quality of life	Employment Household income (In) Satisfaction with life Lives in periphery Pseudo *R*^2^	0.014^**^ 0.008^**^ 0.002 0.019^***^ 0.2704	0.041^***^ 0.019^***^ 0.003 0.025^***^ 0.3713

*Source: SHARE-Israel*.

* *P < 0.1;*

** *P < 0.05;*

*** *P < 0.01*.

While the relative effects of sociodemographic indicators, social network, and health characteristics on the probability of spending out of pocket for healthcare services are similar among those who have supplemental insurance as against those who do not have it, this is not the case where economic resources are concerned. The chances of spending out of pocket for healthcare services are three times greater among employed older adults who have no supplemental health insurance than among employed elders who have such coverage (4.1% points as against 1.4% points). Furthermore, the likelihood of spending out of pocket for healthcare services is 2.5 times greater among those employed older adults who lack supplemental insurance than among the employed older-adult population that has such insurance (1.9% points as against 0.8% point).

The discussion of the third research question is complemented by investigating the predictors of stopping or starting out-of-pocket funding for healthcare services by people in the second half of their lives. To do this, a multivariate analysis using two multinomial models was performed. A multinomial model is a regression that includes a logistic regression allowing more than two discrete results. In other words, the model predicts the probability of different possible results for the distribution of a categorical dependent variable (54).

**Model 1** focuses on the probability of transitions between situations of out-of-pocket expenditure on healthcare services among people aged 50+ in Time 1. The dependent variable is defined within a four-category structure: the individual spends out of pocket for healthcare services at Time 1 and Time 2; spends out of pocket for healthcare services at Time 1 but not at Time 2; does not spend out of pocket for healthcare services at Time 1 but does so later on; and spends nothing out of pocket in either investigation period. To interpret the findings accurately, there must be identity at the point of departure of the various out-of-pocket situations in the first investigation period but not farther on. That is, *the respondents stop spending out of pocket*. The baseline group for the comparison is those who spent out of pocket in both investigation periods.

**Model 2** also centers on the probability of transitions among situations of out-of-pocket spending for healthcare services among persons aged 50+ in the first investigation period, with the dependent variable defined identically to that in the previous model. The difference between the models is the definition of the baseline group. In this model, the baseline group is no out-of-pocket expenditure in either investigation period. Accordingly, the model focuses on respondents who did not spend out of pocket in the first investigation period but did so farther on, i.e., *began to spend out of pocket*.

Estimates of the regression coefficients and their average marginal effects are presented for each model (Table 6). The marginal effect of an explanatory variable denotes the change in the probability of the occurrence of the event that defines the dependent variable (the probability of a transition between situations of out-of-pocket spending), the relevant explanatory variable changing by one unit and the other variables held constant. When the marginal effect is multiplied by 100, the product may be interpreted as a percentage-point change in the probability of the occurrence of the event. For a continuous explanatory variable, the average marginal effect was calculated across all transitions of the explanatory variable, whereas for a dichotomous variable the marginal effect was calculated for the change from value 0 to value 1. Apart from the traditional emphasis in economic research on the marginal effects of explanatory variables on the behavior being investigated, their advantage is that one may use them to compare the absolute sizes of the same variables in different situations of out-of-pocket spending for healthcare services in the multinomial models used in this study. Accordingly, in describing the findings, reference is made to the marginal effects for which, in accordance with their statistical significance, the existence or non-existence of a significant effect of a given variable on out-of-pocket spending is defined.

**Table 6:** Multinomial models for transitions among situations of out-of-pocket expenditure on healthcare services among persons aged 50+ in investigation time 1 (marginal effect).

		**Model 1**	**Model 2**
		**Time 1— out-of-pocket exp.** **Time 2— no out-of-pocket exp. (baseline group— out-of-pocket exp. in both waves)**	**Time 1— no out-of-pocket exp. Time 2— out-of-pocket exp. (baseline group—no out-of-pocket exp. in both waves)**
Sociodemographic characteristics and social network	**Age (baseline: 50–64)**		
	65–79	−0.009^**^	0.008^**^
	80+	−0.009^**^	0.013^**^
	Gender (baseline: female)	0.008	0.014
	Education (years)	0.004*	−0.016^***^
	**Nonimmigrant Jews**		
	Arabs	0.036^**^	−0.020^**^
	FSU immigrants	0.074^***^	−0.022^**^
	Lives with others (baseline: lives alone)	−0.007^*^	0.014^***^
	Size of household	−0.004	−0.006
Health	No. of chronic illnesses	−0.062^***^	0.029^***^
	Increase in no. of chronic illnesses	−0.022^***^	0.019^**^
	Self-rated health	−0.016^**^	0.012^**^
	Worsening in self-rated health	−0.023^***^	0.009^*^
	EURO-D	−0.002	−0.004^***^
Economic resources and quality of life	Employment	−0.011^***^	0.034^***^
	Household income (In)	−0.010^***^	0.006^**^
	Supplemental health insurance	0.006^**^	−0.017^***^
	Lives in periphery	−0.018^***^	0.015^**^
Pseudo *R*^2^		0.2696	0.2412

*Source: SHARE-Israel*.

* *P < 0.1;*

** *P < 0.05;*

*** *P < 0.01*.

### Model 1—Ceasing to Spend Out of Pocket

The results of the marginal-effect estimation indicate that the probability of ceasing to spend out of pocket for healthcare services is negatively dependent on age. The probability of terminating such expenditure correlates positively with education (Each added year of schooling raises the probability of termination by 0.4% point.). The probability of terminating out-of-pocket expenditure is 3.6% points higher among Arabs than among non-immigrant Jews and 7.4% points higher among immigrants than among non-immigrant Jews. Respondents who live with others are 0.7% point less likely to stop spending out of pocket than are those who live alone.

Furthermore, the poorer the respondent's health was in the first investigation period, the less likely it was that he or she would stop spending out of pocket later on. Namely, adding one chronic illness at Time 1 lowers the probability of a person's no longer spending out-of-pocket by 6.2% points. The effect of a decline in state of health due to the addition of one chronic illness during the interval between the waves on the probability of terminating out-of-pocket expenditure is substantially stronger than the effect of having one chronic illness in Time 1: −2.2% points as against −6.2, respectively. A one-point change in self-rated health (on a 1–5 scale) lowers the probability of ceasing to spend out of pocket for healthcare services by 1.6% point. A decline in self-assessed state of health between the two waves, everything else held constant, lowers the probability of terminating out-of-pocket expenditure on healthcare services by 2.3% point.

Finally, employed older adults are less inclined than unemployed older adults to stop spending out of pocket for healthcare services, whereas the effect of household income, an indicator of the household's economic resilience, is found to be negative, i.e., it reduces the probability of ceasing to spend out of pocket for healthcare services. Having supplemental insurance raises the probability of terminating out-of-pocket expenditure on healthcare services; living in a peripheral area acts in the opposite direction.

### Model 2—Starting to Spend Out of Pocket

The results of the marginal-effects estimation indicate that the probability of starting to spend out of pocket for healthcare services is positively dependent on age and negatively correlated with education (Each added year of schooling lowers it by 1.6% points.). The probability of starting to spend out of pocket was 2.0% points lower among Arabs than among non-immigrant Jews and 2.2% points lower among recent immigrants than among non-immigrant Jews. Those who lived with others were 1.4% point more likely to start spending out-of-pocket than were those who lived alone.

The chances of starting to spend out of pocket rose in tandem with poorer health in the first investigation period: The addition of one chronic illness at Time 1 raised the likelihood of beginning to spend out of pocket by 2.9% points; the effect of a decline in state of health due to the addition of one chronic illness between the two waves on the probability of starting to spend out of pocket was 50% lower than the effect of having one chronic illness at Time 1: 1.9% points as against 2.9, respectively. A one-point change in self-rated health (on a 1–5 scale) raised the probability of starting to spend out of pocket for healthcare services by 1.2% points. A decline in self-rated health between the waves, all other factors held constant, increased the likelihood of starting to spend out of pocket for healthcare services by 0.9% point. An increase in the EURO-D index reduced the probability of starting to spend out of pocket for healthcare services by 0.4% point.

Employed older adults are more inclined than unemployed older adults to start spending out of pocket for healthcare services. The effect of household income, an indicator of the household's economic resilience, was found to be positive, meaning that it raised the probability of starting to spend out of pocket for healthcare services. Having supplemental insurance lowered the probability of starting to spend out of pocket for healthcare services, whereas living in a peripheral area acted in the opposite direction.

## Discussion

This study used the SHARE-Israel database to investigate out-of-pocket spending for healthcare services among the elder population in Israel. The findings show that more than 85% of respondents ages 50+ who received healthcare services had to pay some amount out of pocket for them (€ 809 at Time 1; € 832 at Time 2), beyond their fixed contribution for national health insurance and reimbursement of expenses by employers. As for the distribution of the types of out-of-pocket expenditure, more than 80% of respondents who spent out of pocket did so for prescription medicines, whereas the share of those who had to spend out of pocket for care in inpatient settings, in nursing institutions, or at home was two-thirds lower on average (24 and 22%, respectively). These trends persisted after 2 years of follow-up, usually at even higher levels. The findings reinforce what is known about the long-term downward trend in funding of healthcare services by the public healthcare system and the system's need for out-of-pocket funding.

On average, out-of-pocket expenditure on healthcare services was 4.5–5.2% of the total annual income of older adults' households. Importantly, this does not include direct household outlays for supplemental or private health insurance, insofar as such coverage is taken out. The high rate of ownership of supplemental health policies (84% among those who spent out of pocket for healthcare services), the steady increase in households' ownership of commercial insurance policies (55), and the slowing of growth in real household income over the years show that the share of private funding of the healthcare system in total annual household income is trending upward and that its actual rate is even higher than that noted above. The need to fund the healthcare system privately is crimping access to medical services for weak population groups by making it harder for them to afford the rising prices and by making the services themselves less available. It is also exacerbating inequalities in disposable-income distribution and helping to worsen poverty among groups that are weak to begin with (28).

Multivariate analysis of the probability of out-of-pocket funding of healthcare services among those who receive medical care in the second half of their lives yields a comprehensive picture of the factors that affect this probability. Having supplemental health insurance is the strongest determinant of the decision. One may and can attribute this outcome to the regulator's decision to hold the HMOs responsible for delivering the basket of services covered by national health insurance and their ability to deliver additional services within the framework of supplemental insurance (56). This explains why citizens have an economic incentive to pay a premium in order to mitigate the risk to their health and why such a large proportion of households take out such insurance.

The individual's state of health, measured in terms of the number of his or her chronic illnesses, is found to have a unique effect on the aforementioned probability. Furthermore, changes in state of health over time have a significant effect on the probability. This gives an indication of the long-term effect of the health motive on the investment of private resources in funding healthcare services.

The motive of financial and social support that older adults receive from and give to people in their immediate surroundings increases the probability of out-of-pocket funding healthcare services in old age. A possible explanation for this may trace to characteristics of these individuals' social structure and relations with their close surroundings (45, 46).

Analysis of the probability model also shows that the economic resources of the elder and his or her household, measured by the proxy of household employment and income, figure significantly in the decision on whether to spend out of pocket for healthcare services. This decision is also positively dependent on the older adult's age (39, 40) but negatively dependent on his or her level of education. It is also found that the probability of out-of-pocket funding varies among members of different nationalities and between non-immigrant Jews and recent immigrants from the former Soviet Union. A possible explanation for this is that Arab and/or immigrant households are at a disadvantage relative to non-immigrant Jews in sources of income and economic ability to pay for healthcare services not covered by national health insurance. It is also found that the probability of out-of-pocket expenditure on healthcare services is higher among older adults who dwell in peripheral localities than among those who live elsewhere. This outcome emphasizes the inequality that besets the system today (11).

The findings of the model that probed transitions among situations of out-of-pocket expenditure on healthcare services show that employed elders are less inclined to terminate such expenditure and that the effect of household income, an indicator of the household's economic resilience, lowers the probability of ceasing to spend out of pocket for these services. In addition, having supplemental health insurance makes it more likely that an older adult will stop spending out of pocket for healthcare services, whereas living in a peripheral area has the opposite effect. The direction of the results in regard to the onset of out-of-pocket spending for healthcare services is the opposite. These outcomes emphasize all the more the centrality of economic motives in the question of the long-term stability of out-of-pocket expenditure on healthcare services.

As described at length above, the study was based on the use of data from the SHARE survey. When secondary data are used, it is customary to note the limitation that the survey represents only the group that it tests and fails to represent the entire population accurately. In principle, this concern should not be dismissed. In the case at hand, however, the SHARE data were gathered by means of a stratified cluster survey of the older-adult population in Israel, in which each cluster sampled, in a hierarchical manner, comprises people living in households in statistical regions of the strata. After the data were gathered, the demographic characteristics parsed by population groups were compared with parallel data from the Israel Central Bureau of Statistics, and where necessary the sample was corrected by increasing it in the relevant groups. The database thus obtained does accurately represent the entire population.

## Conclusions

This study is the first of its kind in Israel that identifies the predictors of out-of-pocket funding of medical treatments among older adults and infers from them about the predictors of out-of-pocket funding of healthcare services among people in the second half of their lives.

The study stresses that the share of out-of-pocket funding of healthcare in older adult households' total annual income is trending upward. The funding of older-adult healthcare services in Israel is notably unequal. Additionally, economic motives are central in determining whether older adults' out-of-pocket expenditure on healthcare services will stabilize over time. The findings emphasize the need to enhance the healthcare system's awareness of the profile of those aged 50+ who find it necessary to pay out of pocket for healthcare services.

In view of the demographic forecasts, the healthcare system and its policymakers need to become more aware of the profile of those aged 50+ who have to pay out-of-pocket for medical care. The percent increase in ownership of supplemental or commercial health insurance compensates partly for the decision on whether to spend out of pocket. Given the steady increase in the share of older adults who take out health insurance, the healthcare system and the decision-makers should explore alternative ways of protecting publicly funded insurance among the older adult population for the time when they will need it.

## Data Availability Statement

Publicly available datasets were analyzed in this study. This data can be found here: http://www.share-project.org/home0.html.

## Author Contributions

The author confirms being the sole contributor of this work and has approved it for publication.

## Funding

This paper uses data from SHARE Waves 7 and 8 (DOIs: 10.6103/SHARE.w7.711, 10.6103/SHARE.w8.100); see Börsch-Supan et al. (50) for methodological details. The SHARE data collection was funded by the European Commission, DG RTD through FP5 (QLK6-CT-2001-00360), FP6 (SHARE-I3: RII-CT-2006-062193, COMPARE: CIT5-CT-2005-028857, SHARELIFE: CIT4-CT-2006-028812), FP7 (SHARE-PREP: GA N°211909, SHARE-LEAP: GA N°227822, SHARE M4: GA N°261982, DASISH: GA N°283646) and Horizon 2020 (SHARE-DEV3: GA N°676536, SHARE-COHESION: GA N°870628, SERISS: GA N°654221, SSHOC: GA N°823782) and by DG Employment, Social Affairs & Inclusion through VS 2015/0195, VS 2016/0135, VS 2018/0285, VS 2019/0332, and VS 2020/0313. Additional funding from the German Ministry of Education and Research, the Max Planck Society for the Advancement of Science, the U.S. National Institute on Aging (U01_AG09740-13S2, P01_AG005842, P01_AG08291, P30_AG12815, R21_AG025169, Y1-AG-4553-01, IAG_BSR06-11, OGHA_04-064, HHSN271201300071C, RAG052527A) and from various national funding sources is gratefully acknowledged (see www.share-project.org).

## Conflict of Interest

The author declares that the research was conducted in the absence of any commercial or financial relationships that could be construed as a potential conflict of interest.

## Publisher's Note

All claims expressed in this article are solely those of the authors and do not necessarily represent those of their affiliated organizations, or those of the publisher, the editors and the reviewers. Any product that may be evaluated in this article, or claim that may be made by its manufacturer, is not guaranteed or endorsed by the publisher.

## Footnotes

^1^Horev et al. (38) estimate the damage to the Israeli economy on this account at ILS 0.5 billion (approx. USD 125 million) per year.

^2^Out-of-pocket expenditure on any healthcare service includes that incurred for dental services. According to a recently published study, out-of-pocket expenditure on dental services is higher among those of higher class, higher income, and (partially) higher levels of education (51).
